# Prognostic Role of Mid-Regional Proadrenomedullin in Patients Undergoing Emergency Laparotomy: An Exploratory Study

**DOI:** 10.7759/cureus.114064

**Published:** 2026-08-06

**Authors:** Deepak Singla, Sanjay Agrawal, Ranjeeta Kumari, Manisha Naithani, Priya Thayyile Kandy, Shreya Singh, Mishu Mangla

**Affiliations:** 1 Anesthesia and Critical Care, All India Institute of Medical Sciences, Rishikesh, IND; 2 Community and Family Medicine, All India Institute of Medical Sciences, Rishikesh, IND; 3 Biochemistry, All India Institute of Medical Sciences, Rishikesh, IND; 4 Anesthesia and Critical Care, All India Institute of Medical Sciences, Jodhpur, IND; 5 Obstetrics and Gynecology, All India Institute of Medical Sciences, Bibinagar, IND

**Keywords:** biomarkers, lactates, laparotomy, sepsis, sequential organ failure assessment score

## Abstract

Objective: Our primary objective was to determine the ability of mid-regional proadrenomedullin (MR-proADM) levels to predict outcomes, i.e., mortality and clinical cure within 30 days in post-laparotomy patients. The secondary objective was to correlate MR-proADM with other markers of sepsis, such as procalcitonin (PCT) and lactate, and to determine the ability of MR-proADM levels to predict the presence or absence of sepsis on preoperative days 3, 5, and 7 in post-laparotomy patients.

Methods: This was a single-center prospective pilot observational study. Patients ≥18 years of age who underwent emergency laparotomy (EL) under general anesthesia at a tertiary care hospital between January 2022 and July 2023 were enrolled. MR-proADM, lactate, PCT, and sequential organ failure assessment (SOFA) scores were measured pre-operatively and on days 3, 5, and 7. Detection of sepsis, clinical cure, and 30-day all-cause mortality were ascertained.

Results: MR-proADM demonstrated statistically significant correlation with PCT (p<0.001) at all time points, the SOFA score at preoperative, day 5, and day 7, and lactate on day 7 (p<0.001). MR-proADM levels were higher on days 5 and 7 in patients who failed to achieve clinical cure (p=0.0003 and 0.0004, respectively). At all time points, the discriminatory ability of MR proADM to distinguish sepsis from non-sepsis was good. A comparison of MR-proADM levels in patients with 30-day all-cause mortality was performed. The non-survivors had significantly elevated MR-proADM levels at all time points.

Conclusions: MR-proADM is a valuable biomarker that correlates strongly with lactate, PCT, and the SOFA score and can help detect sepsis. Persistently higher values were associated with absence of clinical cure and 30-day all-cause mortality in patients undergoing EL.

## Introduction

According to the Sepsis-3 criteria, sepsis is a dysfunctional host response mounted against an infection that leads to life-threatening organ impairment [1]. Septic shock is a subset of sepsis with affliction of circulatory, cellular, and/or metabolic parameters, posing a greater risk to life when compared to sepsis alone [1]. In a recent study, globally, 48.9 million cases of sepsis were identified, with 11 million fatalities [2]. Hence, prompt sepsis diagnosis, severity assessment, and prognostication become pertinent.

Emergency laparotomy (EL) is performed for life-threatening intra-abdominal pathologies. The presence of preoperative sepsis was associated with a substantial rise in mortality in patients who required EL [3]. The common postoperative complications, such as pneumonia, surgical site infection, and bloodstream infections, were attributed to sepsis. The 9th National Emergency Laparotomy Audit (NELA) conducted in the UK from 2021 to 2023 reported an in-hospital mortality rate of 9.3% [4].

Of late, the role of biomarkers in detecting sepsis has been emphasized. The National Institute of Health defines a biomarker as a value that can be distinctly estimated and used to evaluate either a physiological or pathological process or a pharmacological response to a clinical intervention [5]. Commonly studied biomarkers for sepsis include procalcitonin (PCT), C-reactive protein (CRP), and lactate.

Adrenomedullin is secreted by epithelial cells in response to invading microorganisms [6]. It is secreted as a prohormone called preproadrenomedullin, which is cleaved into three active peptides: adrenomedullin, the amino-terminal peptide of proadrenomedullin (PAMP), and adrenotensin. Between PAMP and adrenomedullin, there is a region with no known activity, called mid-regional proadrenomedullin (MR-proADM) [7]. Although MR-proADM itself has no significant clinical activity, it can be used indirectly to estimate the adrenomedullin levels. Compared to adrenomedullin, MR-proADM has a longer half-life of several hours, and its concentration can be easily analyzed [8].

However, there was a paucity of data on the diagnostic utility of MR-proADM for post-laparotomy patients. So, we aimed to explore the ability of MR-proADM levels to predict the development of outcomes (mortality/clinical cure) within 30 days in post-laparotomy patients. We hypothesized that MR-proADM would predict outcomes in post-laparotomy patients with good accuracy.

## Materials and methods

Study design

It was a single-center prospective exploratory study conducted at a tertiary care hospital from January 2022 to July 2023. It was approved by the Institutional Ethics Committee vide letter no. AIIMS/IEC/20/72 and was registered with the Clinical Trials Registry of India (CTRI/2021/10/037434). We followed the Declaration of Helsinki and the Strengthening the Reporting of Observational Studies in Epidemiology (STROBE) guidelines in reporting our results. Patients >18 years of age who required EL under general anesthesia were recruited. Exclusion criteria were the presence of refractory shock with multi-organ failure at admission, need for re-exploratory laparotomy after primary surgery, and the presence of cystic fibrosis, congestive heart failure, respiratory failure (P/F ratio <100), chronic kidney disease, cirrhosis, active malignancy, or an immunocompromised condition. An immunocompromised condition was defined as one of the following: (a) HIV infection, (b) neutropenia (<500 neutrophils/mm^3^), and (c) active chemotherapy within the three months preceding eligibility or immunosuppressive therapy after organ transplantation.

Informed written consent was obtained from the patient or the surrogate decision maker before enrolment. Relevant demographic, clinical, and lab data were collected. Sequential organ failure assessment (SOFA) scores, lactate, PCT, and MR-proADM were measured preoperatively. An acute increase in SOFA score by 2 or more points from baseline (assumed to be 0 if no prior organ dysfunction is known) along with a suspected or confirmed source of infection was used as the criterion for sepsis. 

There was no interference with the intraoperative anesthesia or surgical technique. SOFA scores, lactate, PCT, and MR-proADM were again measured on postoperative days 3, 5, and 7. An increase in SOFA score by 2 or more from the previous value, with suspected or confirmed source of infection as mentioned above, was used as the criterion for sepsis in the postoperative period. In the postoperative period, the treating surgeon/intensivist was free to initiate antibiotic therapy as per the hospital antibiogram or culture reports, and there was no interference with patient management. The MR-proADM results were not freely accessible to them, but, if desired, test results were made available to them (as mandated by our ethics committee in the interest of patient care). However, in practice, the treating surgeon and intensivist never asked for the MR-proADM results and followed set hospital management protocols. All patients were observed for 30 days postoperatively. 

MR-proADM Estimation Procedure

A 2 mL blood sample was collected in an ethylenediaminetetraacetic acid tube. It was centrifuged at 2000-3000 rpm for 15 minutes. It was frozen to -20°C until further testing. MR-proADM levels were analyzed using the Human MR-proADM GENLISA^TM^ ELISA kit (Krishgen Biosystems, India). It is based on the sandwich enzyme-linked immunosorbent assay (ELISA) principle. The diagnostic range of the kit was 30-480 pg/mL, and the sensitivity was ±7.5 pg/mL.

Our primary objective was to determine the ability of MR-proADM levels to predict outcomes, i.e., mortality and clinical cure within 30 days in post-laparotomy patients. The clinical cure was defined as the resolution of the underlying pathology for which the patient was operated, with no evidence of recurrence, no requirement for redo surgery, discontinuation of antibiotics for >48 hours, and no need for initiating antibiotics for >48 hours after cessation. All-cause 30-day mortality was defined as death due to any cause within 30 days after an index EL. The secondary objectives were to correlate MR-proADM with the SOFA score and other markers of sepsis, such as PCT and lactate, and to determine the ability of MR-proADM levels to predict the presence or absence of sepsis in preoperative and on days 3, 5, and 7 in post-laparotomy patients.

Sample size estimation

The effect size was calculated using the effect size converter (made by Lin H. Code released under the MIT license. https://www.escal.site), based on a similar study in which the area under the curve (AUC) of MR-proADM was 0.76 [9]. Based on these values, the effect size was 0.999. Using G*Power software, version 3.1, with an alpha error of 0.05 and a power of 80%, the sample size was calculated as 14 tests in each group (survivors and non-survivors). So, a minimum of 14 tests was to be performed in each group to estimate the AUC.

Statistical analysis

Data analysis was performed in the Statistical Package for Social Sciences (SPSS) version 23.0 (IBM Corp., Armonk, NY). The first 24 hours of the hospital stay after laparotomy were considered day 1, and events up to 30 days were analyzed. Independent sample t-test or Mann-Whitney U test was used for continuous variables. The Pearson correlation coefficient was used to study the correlation of MR-proADM with PCT, lactate, and the SOFA score. Accuracy and predictive values of biomarkers were calculated using the area under the receiver operating characteristic curve (AUROC). A p-value of 0.05 was considered statistically significant.

## Results

**Figure 1:**
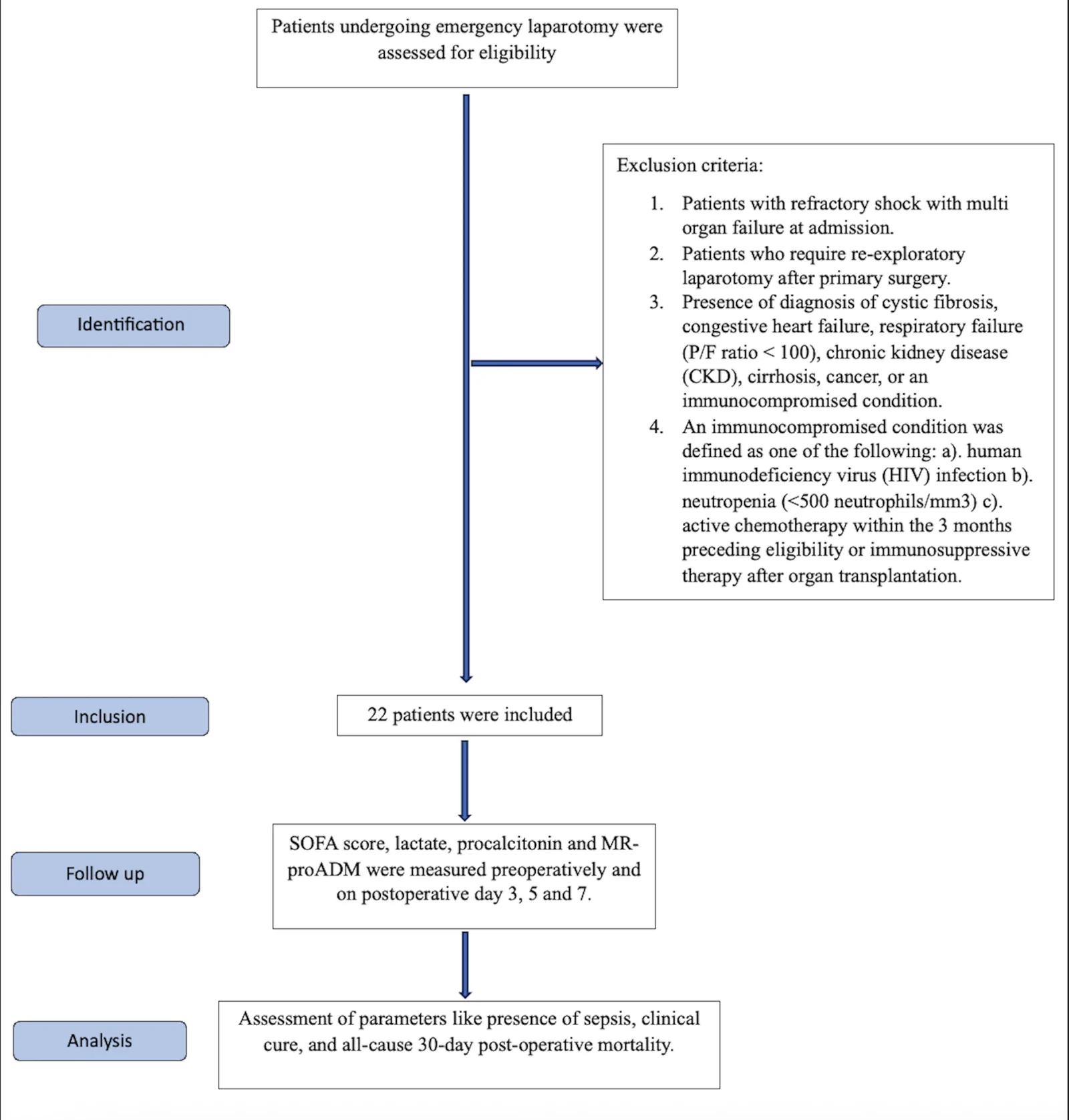
Patient flow diagram MR-proADM, mid-regional proadrenomedullin; SOFA, sequential organ failure assessment.

Twenty-two patients were enrolled in the study from January 2022 to July 2023. Two patients were discharged on day 6. Out of 22 patients, 4 did not achieve clinical cure within 30 days; among those, 3 patients died (Figure 1).

The mean ± SD age of the study population was 49.9 ± 15.2, the male-to-female ratio was 13:9, and BMI was 23.6 ± 3.2 kg/m^2^. 2 (9.1%) patients were American Society of Anesthesiologists (ASA) Class I E, 1 (4.5%) was ASA Class II E, and 19 (86.4%) were ASA Class IV E. In terms of comorbidities, 5 (22.7%) patients had diabetes mellitus, 7 (31.8%) had hypertension, 3 (13.63%) had asthma, 2 (9.1%) had coronary artery disease, and 2 (9.1%) had hypothyroidism.

In the preoperative period, 19 patients had sepsis while 3 did not have sepsis. The mean SOFA score in patients with sepsis was 3.42, and in patients with no sepsis it was 1 (p=0.006) (Table 1).

**Table 1:** Mean (SD) levels of biomarkers and SOFA score in patients with and without sepsis at different time points 1, independent sample T-test; 2, Mann-Whitney U test. df: degrees of freedom; MR-proADM, mid-regional proadrenomedullin; SD, standard deviation; SOFA, sequential organ failure assessment.

Preoperative	Sepsis present (19)	Sepsis absent (3)	Static	df	p-value
MR-proADM	179.02 (88.73)	122.71 (34.985)	1.07^1^	20.00	0.298^1^
Procalcitonin	11.75 (15.17)	1.54 (0.974)	11.00^2^	20.00	0.108^2^
Lactate	3.92 (1.46)	1.87 (0.306)	2.38^1^	20.00	0.028^1^
SOFA score	3.42 (1.35)	1.00 (0.00)	0.00^2^	20.00	0.006^2^
Day 3	Sepsis present (10)	Sepsis absent (12)			p-value
MR-proADM	205.53 (124.48)	148.95 (73.07)	1.33^1^	20.00	0.199^1^
Procalcitonin	14.26 (17.36)	7.22 (7.94)	50.00^2^	20.00	0.539^2^
Lactate	3.74 (1.16)	3.65 (2.86)	43.50^2^	20.00	0.291^2^
SOFA score	5.30 (1.57)	2.33 (1.30)	4.85^1^	20.00	<0.001^1^
Day 5	Sepsis present (3)	Sepsis absent (19)			p-value
MR-proADM	354.96 (74.49)	140.93 (57.08)	-5.83^1^	20.00	<0.001^1^
Procalcitonin	44.26 (2.01)	4.03 (4.00)	0.00^2^	20.00	0.001^2^
Lactate	5.80 (3.12)	2.79 (1.55)	-2.74^1^	20.00	0.013^1^
SOFA score	9.33 (1.53)	2.58 (1.39)	-7.76^1^	20.00	<0.001^1^
Day 7	Sepsis present (3)	Sepsis absent (17)			p-value
MR-proADM	328.81 (40.11)	127.82 (67.22)	-4.96^1^	18.00	<0.001^1^
Procalcitonin	49.87 (3.13)	3.15 (2.05)	-34.02^1^	18.00	<0.001^1^
Lactate	6.83 (4.51)	2.26 (1.14)	-.3.95^1^	18.00	<0.001^1^
SOFA score	12.33 (1.53)	2.18 (1.13)	-13.72^1^	18.00	<0.001^1^

There were no significant differences in the levels of MR-proADM, PCT, and lactate between patients with sepsis and those without at presentation and on day 3 (Table 1). On day 5, MR-proADM levels were reported to be significantly raised in patients with sepsis (p<0.001). PCT also showed a statistically significant difference between the two groups (p=0.001). However, there was no statistically significant difference in lactate levels between the two groups. On day 7, all three biomarkers showed statistically significant differences (p<0.001) between the two groups (Table 1).

The correlation of MR-proADM with PCT, lactate, and the SOFA score was studied using Pearson correlation in Table 2.

**Table 2:** Correlation of MR-proADM with procalcitonin, lactate, and the SOFA score MR-proADM, mid-regional proadrenomedullin; SOFA, sequential organ failure assessment.

Procalcitonin	Pearson correlation	p-value	95% confidence interval	
			Lower	Upper
At Preop	0.777	<0.001	0.53	0.90
At Day 3	0.865	<0.001	0.70	0.94
At Day 5	0.860	<0.001	0.69	0.94
At Day 7	0.762	<0.001	0.51	0.91
Lactate	Pearson correlation	p-value		
At Preop	0.162	0.473	-0.28	0.55
At Day 3	0.173	0.441	-0.27	0.55
At Day 5	0.311	0.159	-0.13	0.65
At Day 7	0.47	0.037	0.03	0.75
SOFA score	Pearson correlation	p-value		
At Preop	0.31	0.158	-0.13	0.65
At Day 3	0.44	0.040	0.02	0.73
At Day 5	0.611	0.003	0.26	0.82
At Day 7	0.74	<0.001	0.44	0.89

We found MR-proADM level to strongly correlate with the levels of PCT at all time points, demonstrated a moderate correlation with lactate on day 7 (r=0.47, p=0.037), moderate correlation with SOFA score at day 3 (r=0.44, p=0.040), and a strong and statistically significant correlation on day 5 (r=0.611, p=0.003) and day 7 (r=0.762, p<0.001) (Table 2). 

On the preoperative day, MR-proADM had acceptable discriminatory ability to distinguish sepsis from non-sepsis, with an AUC of 0.702 and excellent sensitivity (100%) but low specificity (47.37%) (Table 3).

**Table 3:** Sensitivity, specificity, positive and negative predictive values, and accuracy of MR-proADM for detecting sepsis at different time points AUC, area under the curve; MR-proADM, mid-regional proadrenomedullin.

	Preoperative (n=22)	Day 3 (n=22)	Day 5 (n=22)	Day 7 (n=20)
AUC	0.702	0.642	0.982	1
Cut-off (Youden’s index)	163.585	175.28	252.715	286.825
Sensitivity (%)	100	83.33	94.74	100
Specificity (%)	47.37	60	100	100
Positive predictive value (%)	23.08	71.43	100	100
Negative predictive value (%)	100	75	75	100
Accuracy (%)	54.55	72.73	95.45	100

By day 7, there was perfect discriminatory ability to distinguish sepsis from non-sepsis, with an AUC of 1 and an optimal cut-off of 286.825 pg/mL, with sensitivity, specificity, positive predictive value, negative predictive value, and accuracy all at 100%. However, as the data include only 3 patients with sepsis and 17 patients without, this is an unstable exploratory estimate with high overfitting risk.

Eighteen patients had clinical cure, while four did not have resolution of disease. MR-proADM levels were elevated in patients without clinical cure. At the preoperative time point, this difference was not statistically significant (p=0.0521). But it was statistically significant at all postoperative time points (Table 4).

**Table 4:** Mean MR-proADM levels with clinical cure within 30 days and all-cause 30-day mortality 1, independent sample T-test. df: degrees of freedom; MR-proADM, mid-regional proadrenomedullin.

MR-proADM	Clinical cure, yes (n=18)	Clinical cure, no (n=4)	Static (t-value)	df	p-value	95% confidence interval	
						Lower	Upper
At Preop	154.89 ± 76.22	245.35 ± 94.58	-2.06	20.00	0.0521^1^	-0.01	2.27
At Day 3	147.90 ± 68.71	295.11 ± 146.59	-3.13	20.00	0.0053^1^	0.51	2.92
At Day 5	139.83 ± 58.53	306.40 ± 114.59	-4.31	20.00	0.0003^1^	1.05	3.67
At Day 7	124.11 ± 67.6	293.41 ± 78.01	-4.36	18.00	0.0004^1^	1.06	3.77
MR-proADM	Mortality, no (n=19)	Mortality, yes (n=3)			p-value		
At Preop	153.16 ± 74.46	286.53 ± 56.96	-2.94	20.00	0.008^1^	-3.15	-0.47
At Day 3	145.86 ± 67.37	357.11 ± 95.75	-4.81	20.00	<0.001^1^	-4.49	-1.44
At Day 5	140.93 ± 57.08	354.96 ± 74.49	-5.83	20.00	<0.001^1^	-5.25	-1.95
At Day 7	127.82 ± 67.22	328.81 ± 40.11	-4.96	18.00	<0.001^1^	-4.66	-1.49

A comparison of MR-proADM levels in patients with 30-day all-cause mortality was performed. Nineteen patients were survivors, and three were non-survivors. The non-survivors had elevated MR-proADM levels (Table 4).

## Discussion

We conducted a study to determine the correlation of MR-proADM with other markers of sepsis in patients undergoing EL under general anesthesia. The cohort consisted of predominantly middle-aged patients. Since 19 (86.4%) patients had preoperative sepsis, the patients were classified as ASA PS-IV. The MR-proADM levels were elevated in patients with sepsis compared with those without sepsis, although this difference did not reach statistical significance preoperatively and on day 3. However, on days 5 and 7, the MR-proADM levels were significantly elevated in patients with sepsis. MR-proADM levels were strongly correlated with PCT at all time points, with lactate on day 7 (r=0.47, p=0.037), and showed a moderate correlation with the SOFA score on day 3 (r=0.44, p=0.040), day 5 (r=0.611, p=0.003), and day 7 (r=0.762, p<0.001). The MR-proADM levels were significantly elevated in patients who did not achieve clinical cure on days 3, 5, and 7, and those who had mortality at all time points. 

We studied MR-proADM because it was more precise than conventional markers, such as CRP and PCT, for predicting outcomes and mortality in patients with sepsis admitted to the ICU. A combination of PCT and MR-proADM has been shown to enable early identification of organ failure in sepsis [10]. It was not only an early indicator of sepsis but also a predictor of 28-day and 90-day mortality among non-survivors [11].

We found significantly elevated MR-proADM levels on days 5 and 7 in post-laparotomy patients with sepsis. Though the mean levels were higher preoperatively and on day 3, the difference was not statistically significant. A small sample size and varied indications for laparotomy might have resulted in non-significant findings at these time points. Our observations are consistent with the findings of Spoto et al., who studied 209 patients with sepsis and 50 controls presenting to the emergency department. MR-proADM levels were elevated in sepsis, 2.55 nmol/L, compared with controls, 1.19 (p<0.0001) [12].Rueda et al. conducted a prospective multicenter study in 370 patients undergoing abdominal onco-surgery. In multivariate analysis, an elevated preoperative MR-proADM level ≥0.70 nmol/L was associated with the postoperative requirement of organ support [13]. The drawback of these studies is that MR-proADM levels were measured only once. We have addressed this gap by estimating the serial trends. This provided insights into the evolving pathophysiological course, thus aiding in therapy. 

MR-proADM demonstrated statistically significant correlation with PCT at all time points and with lactate on day 7. Regarding the SOFA score, there was a statistically significant correlation at all time points except on the preoperative day, where the correlation was not statistically significant. Consistent with our findings, Christ-Crain et al. conducted a prospective study in 101 critically patients and 160 age-matched controls, MR-proADM levels on admission highly correlated with PCT (r=0.65, p<0.001) and variable strength of correlation with other markers such CRP (r=0.41, p<0.001), APACHE-II (r=0.42, p<0.001), SAPS II (r=0.4, p<0.001), serum IL-6 (r=0.53, p<0.001) [14]. Wadie et al. studied 30 patients with sepsis and found that MR-proADM correlated with levels of the SOFA score (r=0.549, p=0.002) and APACHE-II (r=0.547, p=0.002) [15]. A recent meta-analysis comparing presepsin and MR-proADM for diagnosing sepsis and septic shock found MR-proADM to have a significantly higher accuracy than presepsin [16].

At all time points, the discriminatory ability of MR proADM to distinguish sepsis from non-sepsis was good. A comparison of MR-proADM levels in patients with 30-day all-cause mortality was performed. The non-survivors had significantly elevated MR-proADM levels at all time points. A recent systematic review and meta-analysis, including 2038 patients, found MR-proADM to have a high sensitivity of 83% and a specificity of 90% for the diagnosis of sepsis [17]. The AUC was found to be 0.91, and the optimal cut-off value was 1-1.5 nmol/L. A study using MR-proADM for diagnosing sepsis after cardiac surgery found an AUC of 0.88, an optimal cut-off of 5.1 nmol/L, with a sensitivity of 78.5% and a specificity of 85% [18]. Another study using MR-proADM to identify spontaneous bacterial peritonitis in patients with cirrhosis found it to have a high diagnostic performance, with an AUROC of 0.746 at an optimal cut-off of ≥2.50 nmol/L. Further adding MR-proADM with Child-Pugh score and an absolute polymorphonuclear neutrophil count ≥250 cells/mm3 will increase the AUROC to 0.993 [19].

In our study, the MR-proADM levels were significantly elevated in patients who did not achieve clinical cure on days 3, 5, and 7 and those who had mortality at all time points. Similar findings were reported by Valenzuela-Sánchez et al. in a prospective single-center observational study of 120 patients with suspected severe sepsis admitted to the ICU. They observed that on day 5, MR-proADM levels were 3.4 nmol/L in non-survivors versus 1.3 nmol/L in survivors (p=0.0006). Overall levels were 2.4 nmol/L in non-survivors versus 1.6 nmol/L in survivors (p=0.04) [20]. A study conducted by Buendgens et al. in critically ill ICU patients found that a cut-off MR-proADM level of 1.4 nmol/L on the day of admission was associated with an increase in ICU mortality (OR: 3.15, 95% CI: 1.08-9.20, p=0.036). There was also a rise in overall mortality on two-year follow-up (OR: 2.4, 95% CI: 1.12-5.34, p=0.026) [21]. In a study by Cander et al, mortality was higher in patients with increased MR-proADM than in those with lower levels (p<0.05) [22]. These results suggest that MR-proADM can be used as a dynamic marker to monitor the progression of sepsis over time. 

Our study had certain limitations. Firstly, the sample size was small. We could recruit only 22 patients. That was due to the unavailability of additional ELISA kits. The study was conducted at a single center, which may have limited generalizability. Further, we used an ELISA-based assay to measure MR-proADM levels, which has relatively lower sensitivity and specificity compared to time-resolved amplified cryptate emission-based tests. So, future research should aim for multicentric validation with a larger, more diverse patient cohort. Evaluating MR-proADM along with other biomarkers and clinical scores could provide an integrated approach to sepsis detection and antibiotic stewardship.

## Conclusions

Although MR-proADM did not significantly differentiate patients with sepsis from those without sepsis among those undergoing EL due to varied presentations and a small sample size, it demonstrated a strong correlation with sepsis severity, clinical cure, and 30-day mortality. Despite the small sample size of our study, we suggest using MR-proADM as an adjunct biomarker for early risk stratification and prognostication in surgical sepsis. However, larger multicenter studies are needed to validate its role.
